# Undifferentiated In Vitro Cultured *Actinidia deliciosa* as Cell Factory for the Production of Quercetin Glycosides

**DOI:** 10.3390/plants10112499

**Published:** 2021-11-18

**Authors:** Stefano Negri, Sofia Gambini, Stefania Ceoldo, Linda Avesani, Mauro Commisso, Flavia Guzzo

**Affiliations:** Department of Biotechnology, University of Verona, Strada Le Grazie, 15, 37134 Verona, Italy; stefano.negri@univr.it (S.N.); sofia.gambini@univr.it (S.G.); stefania.ceoldo@univr.it (S.C.); linda.avesani@univr.it (L.A.)

**Keywords:** quercetin, flavonoids, plant cell and tissue culture, *Actinida deliciosa*, *Actinidia chinensis*

## Abstract

Land plants produce a vast arsenal of specialized metabolites and many of them display interesting bioactivities in humans. Recently, flavonol quercetin gained great attention in the light of the COVID-19 pandemic because, in addition to the anti-inflammatory, antiviral and anti-cancer activity already described, it emerged as possible inhibitor of 3CLpro, the major protease of SARS-CoV-2 virus. Plant cell and tissue culture (PCTC) is an attractive platform for the biotechnological production of plant metabolites. This technology allows a large amount of water and agricultural land to be saved and, being free of contaminants in the process, it is suitable for scaling up the production in bioreactors. In a project aimed to generate and screen in vitro plant cells for the production of valuable specialized metabolites for commercial production, we generated various cell lines from *Actinidia deliciosa* (kiwi fruit tree) and *Actinidia chinensis* (gold kiwi fruit tree), that were able to produce relevant amounts of quercetin derivatives, mainly quercetin glycosides. Three cell lines from *A. deliciosa* were characterized by targeted and untargeted metabolomics. In standard growing conditions, they produce and accumulate up to 13.26 mg/100 g fresh weight (419.76 mg/100 g dry weight) of quercetin derivatives. To address future industrial applications, these cell lines should be entered into an acceleration program to further increase the amount of these metabolites by optimizing the culture conditions and elicitation.

## 1. Introduction

Land plants are the richest source of novel bioactive natural products for development as drugs, nutraceuticals, cosmetics and agrochemicals. Plants produce diverse “specialized” or “secondary” metabolites to overcome biotic and abiotic stress and to communicate with other organisms. Many of these metabolites display beneficial bio-activities that are important to humans. Thus, plants provide a rich source of bioactive and valuable small molecules. This reflects the immense and unmatched chemical diversity of plants, which is enhanced by their complex secondary metabolic pathways and the high selectivity (regio-selectivity and stereo-selectivity) typical of plant enzymes.

The potential social and economic benefits of this diverse chemical arsenal are very high, particularly in the context of food supplements, nutraceuticals, drug development, the health and wellness industry, and agriculture. Undifferentiated in vitro cultured plant cells and tissues sometimes retain many of the biosynthetic abilities of the original explants, and can rapidly grow to produce a large amount of biomass in a short time, in simple synthetic media containing salts, sugar, vitamins and phytohormones, mainly auxins and cytokinins. Such technology, commonly referred to as “plant cell and tissue culture” (PCTC), offers an attractive and sustainable platform for the production of plant-specialized metabolites in bioreactors. The main advantages are (i) season-independent plant biomass growth; (ii) saving water resources; and (iii) avoiding the use of herbicides and pesticides [1]. Moreover, PCTC is the only alternative for the production of specialized metabolites from rare or endangered plant species, especially when market demand exceeds the availability of plant material or the total synthesis is difficult to obtain. The typical example comes from paclytaxel (taxol), a metabolite accumulated at low concentration in the bark of mature *Taxus brevifolia* and *Taxus baccata* trees and related species. Once that paclitaxel efficacy against the ovarian cancer became clear, there was a sudden sharp increase in the demand for taxol. This raised the concerns on the possible ecological impact of intensive tree collection, causing the so-called “taxol supply crisis” [2].

Since plant cell cultures can be established starting from a very small amount of living material, this can be considered a sustainable technique that reduces the ecological problems caused by the extensive collection of wild plants. Additionally, means that agricultural land which must be dedicated to the growth of food plants is not used.

Many types of metabolites have been observed in cell cultures, including polyphenols. These represent a wide-occurring family of phytochemicals with well recognized beneficial effects on human health and organized in the subclasses of flavonoids, stilbenes, hydroxycinnamic acids, chalcones, dihydrochalcones, ellagic acid and ellagitannins, phenolic acids, coumarins and lignans [3].

In particular, flavonoids can be further distinguished into flavonols, flavanols (and their polymeric forms, i.e., procyanidins), flavanones, flavones, isoflavones and anthocyanins. They all exhibit two aromatic rings (called A and B) joined by a heterocyclic ring (C) containing oxygen. These three rings can be characterized by the presence of methyl, methoxy or hydroxyl groups, which, in turn, can be further linked to sugars and acylated sugars.

The flavonol quercetin features a hydroxyl group bonded to carbon 3 of the C ring and four other hydroxyls bonded to the A and B rings, and is characterized by many recognized properties. Mainly as a consequence of its high antioxidant and ROS-scavenging activities and thanks to its ability to modulate specific pro- and anti-inflammatory signaling pathways and mediators in vivo [4,5], quercetin helps to counterbalance the oxidative processes involved in many inflammation-related pathologies, such as cardiovascular [6] and neurodegenerative diseases, including Alzheimer’s [7,8,9]. Interestingly, by exerting direct proapoptotic activity on cancer cells, quercetin can inhibit the growth of several tumors (including those affecting the breast and colon), making it potentially useful as a synergic treatment combined with traditional chemotherapy [10]. Moreover, quercetin is reported to protect against gastric ulcers, allergies, diabetes and bacterial and viral infections [11,12].

Very recently, against the backdrop of the COVID-19 pandemic, in silico and in vitro evidence confirmed the potent inhibitory activity of quercetin against 3CLPro, the main SARS-CoV-2 protease and rhACE2, the human receptor that is the key to the entrance of the virus into the host cells [13,14]. Several authors, thus, endorsed the exploitation of this flavonol, together with other natural products showing high SARS-CoV-2 inhibition, as a complement to the reference therapy against COVID-19 [15,16]. This triggered the research at a further stage and several clinical trials have been launched to test the beneficial effects of the oral administration of quercetin, either alone or coupled with other supposed synergic compounds (e.g., ascorbic acid [17]), in COVID-19 patients under clinical treatment. To date, one study positively demonstrates that patients receiving COVID-19 therapy, together with a 30 day-supplementation of 1000 mg of Quercetin Phytosome^®^ at the early stages of the disease, developed less severe respiratory symptoms and a reduction in the time taken to obtain a negative test after a positive one [18].

In general, quercetin-based therapeutic interventions (e.g., for the treatment of hypertension or for testing on COVID-19) requires a daily amount of 200–1200 mg. To supply the increasing demand of quercetin for therapeutic use as well as for the supplement industry, PCTC and heterologous production in engineered microbes [19,20,21,22] would represent two promising biotechnological approaches, in parallel to current extraction and purification from rare natural-rich sources (e.g., the flower buds of *Sophora japonica*). In fact, although quite frequent in plants, including edible plants as elderberry and other berries, capers, cloves, Mexican oregano (*Lippia graveolens*), onions and shallots, tea, wine, plum, black olive, asparagus, buckwheat (*Fagopyrum esculentum*) [23,24,25], quercetin accumulates at rather low quantities in comparison to other specialized metabolites, such as anthocyanins and hydroxycinnamic acids. Moreover, flavonoids accumulate, in general, at low levels in plant cell cultures. Probably for this reason, while anthocyanins and hydroxycinnamic acids (e.g., caffeic acid) together with their derivatives (e.g., echinacoside, rosmarinic acid, teupolioside, verbascoside), have been already exploited for industrial production [1,26,27], exploitation of quercetin or other individual flavonoids by PCTC has never been reported.

Within a project aimed to generate and screen plant cell lines for the production of valuable metabolites, three cell lines generated from *Actinidia deliciosa* (kiwifruit tree) showed the ability to accumulate good amounts of several quercetin glycosides. These cell lines were characterized by targeted and untargeted metabolomics to qualitatively and quantitatively define their quercetin profile.

## 2. Results

### 2.1. Establishment of Actinidia Cell Lines

For the establishment of the cell cultures, apical buds, young stem internodes, young nodes with lateral meristem and young leaves of *A. deliciosa* (Ad) and *A. chinensis* (Ac) were explanted in solid medium A. Young internodes of both species were also explanted in medium B, differing depending on the hormonal composition (see Section 4.1).

Two weeks after the initiation of the cultures, all the explants appeared swollen, and showed a small amount of callus. At this stage, explants were cut again in 3–4 pieces, to stimulate callus formation.

**Figure 1:**
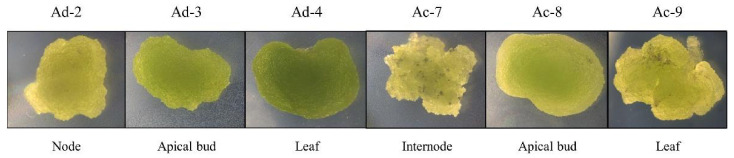
Cell lines of *A. deliciosa* (Ad) and *A. chinensis* (Ac); the plant organ from which each cell culture was initiated is shown.

Three months after the initiation of the cultures, all the explants formed abundant proliferating callus. In medium A, the explants of both species, with the sole exception of the leaves, formed a fast growing light green friable callus. On the other hand, the explanted leaves formed heterogeneous calli, showing both dark green hard and light green soft morphologies. In medium B, all the calli were dark green and hard, undergoing frequent browning. At this stage, medium B was replaced with medium C. Light green and friable calli were continuously selected from the heterogeneous leaf-derived calli. Nine months after the initiation of the culture, various fast-growing Actinidia cell lines were well established. Calli from induction medium A were all friable, while those generated in medium B were hard. Six friable calli were chosen for further characterization (Figure 1). The Ad-2,3,4 and Ac-7,8,9 calli were also put in liquid medium (medium A without agar), where they disaggregated quickly and generated a fine suspension culture.

### 2.2. Untargeted Metabolomics Analysis

Light green friable calli of *A. deliciosa* (Ad-2,3,4) and *A. chinensis* (Ac-7,8,9) were analyzed with an untargeted metabolomics approach. The methanolic extracts were prepared, diluted as described in the Material and Methods section and analyzed by Ultra Performance Liquid Chromatography—PhotoDiode Array—ElectroSpray Ionization—Quadrupole quadrupole Time of Flight (UPLC-PDA-ESI-QqTOF). Since the hard calli are not suitable for growing in a bioreactor in liquid medium, the cell lines obtained on medium B were not further characterized.

**Figure 2:**
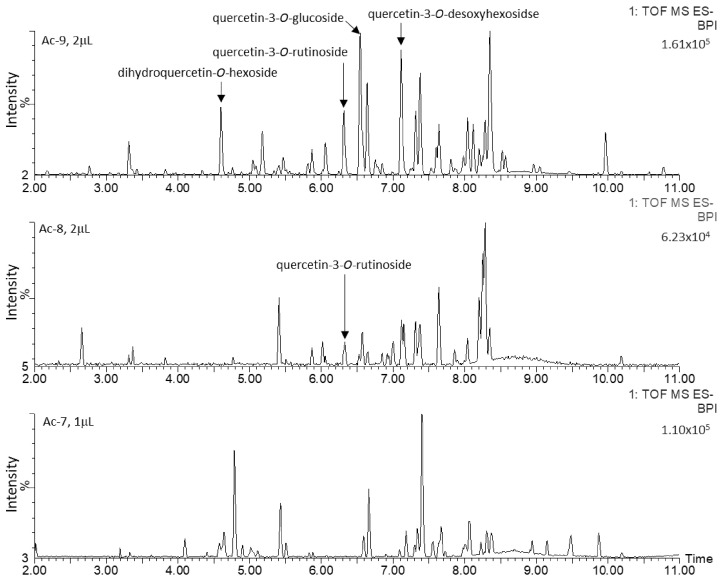
Base Peak Chromatograms (BPC) of 30-day-old calli of Ac-7, Ac-8 and Ac-9 lines, obtained by UPLC-PDA-ESI-QqTOF; these profiles were obtained from different volumes of methanol extracts (1 µL for Ac-7 and 2 µL for Ac-8 and Ac-9).

**Figure 3:**
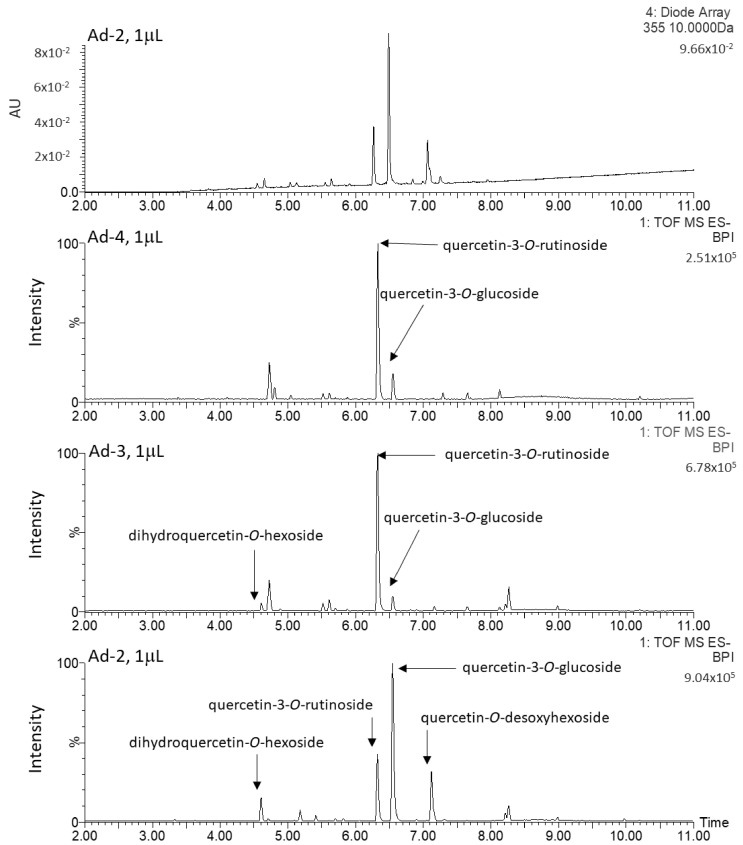
Chromatogram recorded at 355 nm of wavelength and Base Peak Chromatograms (BPC) of 30 days old calli of Ad-2, Ad-3 and Ad-4 lines, obtained by UPLC-PDA-ESI-QqTOF. AU = Absorbance Units.

**Figure 4:**
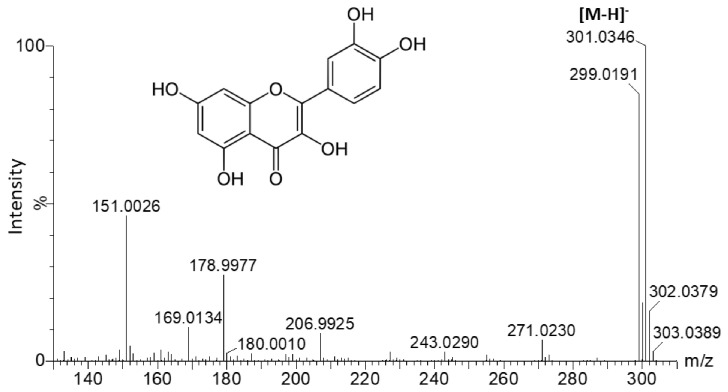
MS spectrum reporting the *m*/*z* diagnostic fragments of the quercetin aglycon.

The chromatographic profiles of the methanolic extracts from the six Actinidia cell lines grown on medium A are shown in Figure 2 and Figure 3. These were characterized and, interestingly, quercetin glycosides represented the major metabolites in Ad lines. Quercetin-3-*O*-glucoside and quercetin-3-*O*-rutinoside were uniquely identified by comparing the retention time, mass to charge ratio (*m*/*z*), isotopic pattern, fragmentation pattern (ms/ms) and UV/Vis absorbance spectrum to those of the authentic standards. The other quercetin derivatives were putatively identified on the basis of their *m*/*z*, absorbance peak at 355 nm of wavelength and the presence of the following diagnostic fragments of the quercetin aglycone: 301.0346, 299.0191, 151.0026 and 178.9977 (Figure 4). 

**Figure 5:**
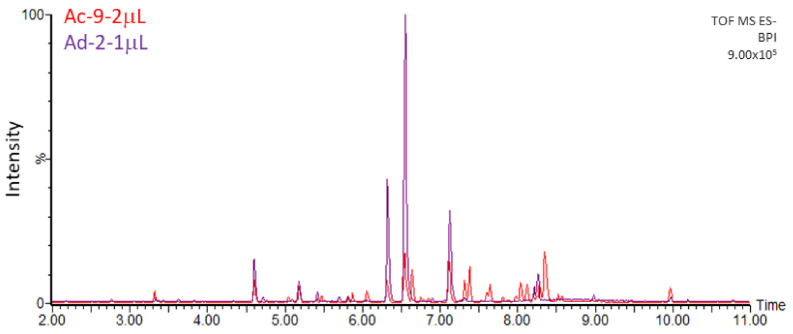
Comparison of chromatograms from 1 µL of methanol extract of Ad-2 (in violet) and 2 µL of Ac-9 (in red), using the same scale in the Y axis (intensity, expressed as percentage of the more intense peak of Ad-2).

The cell lines of *A. chinensis* (Ac) showed complex chromatographic profiles, characterized by many peaks (Figure 2). On the contrary, A. deliciosa (Ad) chromatographic profiles were more simple, characterized by few and very high peaks (Figure 3). The intensities of these signals were not comparable with those presented in Ac, even when 2 µL of Ac samples were injected (Figure 5).

### 2.3. Characterization and Quantification of Quercetin Derivatives in Ad Cell Lines

**Figure 6:**
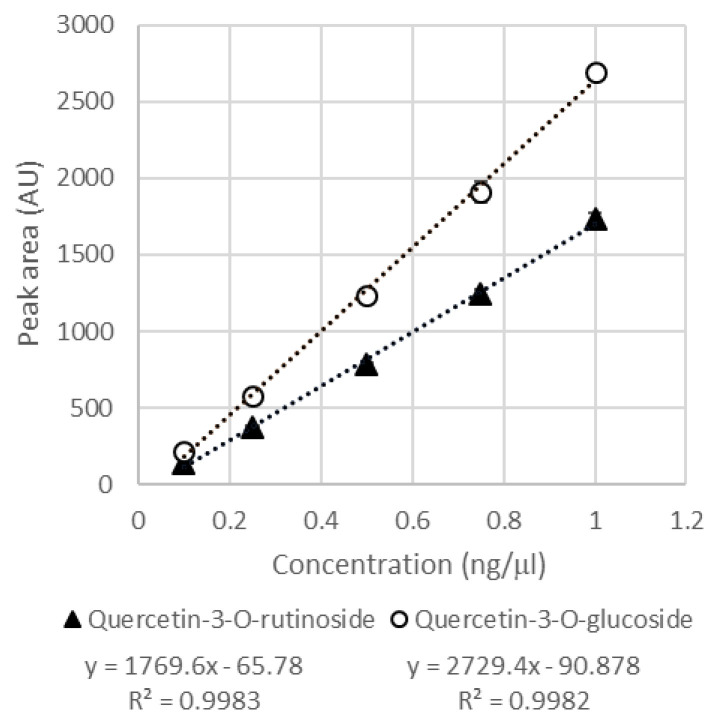
Calibration curve of quercetin-3-*O*-glucoside and quercetin-3-*O*-rutinoside; values are mean of two independent measures, +/− standard deviation. The linear regression equation and the R^2^ are reported.

Since the Ad cell lines produced few and very intense peaks, these lines were considered the best for setting up a quercetin production process. In fact, the presence of a few abundant molecules could greatly simplify the downstream processes of extraction and purification of metabolites. For these reasons, the Ad lines have been further characterized. In Table 1, the features detected by UPLC-PDA-ESI-QqTOF were putatively identified and they included fifteen different quercetin derivatives. Their quantification in Ad cultures was carried out by using only the UPLC-PDA and injecting different amounts of the two commercial standards quercetin-3-*O*-glucoside and quercetin-3-*O*-rutinoside. The peak areas measured at 355 nm wavelength were used to draw the calibration curves (Figure 6).

The calibration curves showed a good linear trend (R^2^ = 0.9982 for quercetin-3-*O*-glucoside and R^2^ = 0.9983 for quercetin-3-*O*-rutinoside). Then, the chromatographic areas at 355 nm wavelength of the quercetin derivatives present in the samples were measured and compared with the calibration curve. The two main metabolites, i.e., quercetin-3-*O*-glucoside and quercetin-3-*O*-rutinoside, were quantified with their own authentic standard, while the other quercetin derivatives were expressed as the equivalent of quercetin-3-*O*-glucoside (Table 2).

### 2.4. Antioxidant Activity of Ad Lines and Correlation with Their Metabolic Profile 

**Figure 7:**
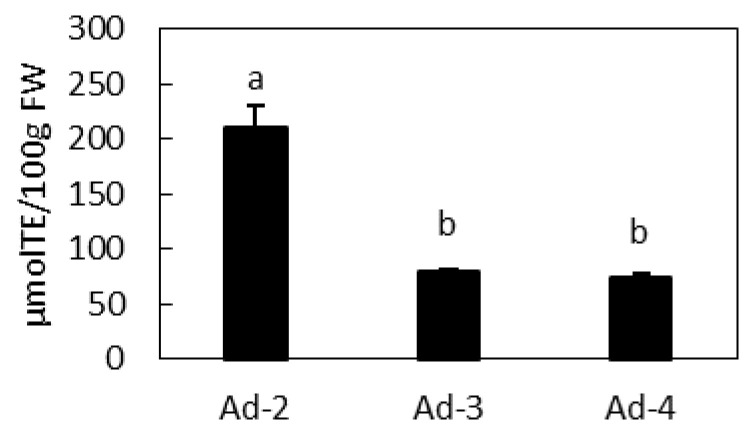
Antioxidant activity of Ad cell lines. TE, Trolox equivalents. Data are means ± standard deviations of *n* = 3. Different letters on bars indicate that means are significantly different according to post hoc SNK-ANOVA (*p* < 0.01).

In order to better characterize the cell lines, their antioxidant activity was explored by FRAP assay as shown in Figure 7. In Ad lines, Ad-2, which accumulates the highest amount of quercetin derivatives, also shows the highest antioxidant activity.

**Figure 8:**
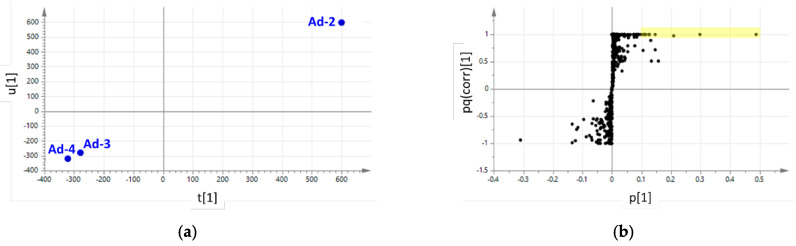
Supervised OPLS analysis of the UPLC-ESI-QqTOF data matrices for Ad cell lines to investigate the relationship between the antioxidant activity and the metabolomics profiles. (**a**) t[1] versus u[1] score plot; (**b**) p[1] versus pq(corr)[1] loading plot; metabolites are represented by black dots; yellow box highlights metabolites with p[1] > 0.1 and pq(corr)[1] > 0.97 (a list is reported in Table 3).

The correlation between the complete metabolomics dataset and antioxidant activity of Ad lines was explored by OPLS analysis. In Figure 8, the t[1] versus the u[1] score plot and the p[1] versus the pq(corr)[1] loading plot are shown. The score plot (Figure 8a) reveals a linear relationship between antioxidant activity (obtained by FRAP, Y variable) and the metabolites (X variables). The higher antioxidant activity of Ad-2 correlates with the metabolites highlighted in the loading plot (Figure 8b), and the metabolites with high p and high pq(corr) (i.e., abundant metabolites with high correlation with antioxidant activity) are shown in Table 3. These metabolites include quercetin-3-*O*-rutinoside and other quercetin derivatives.

## 3. Discussion

In a project aimed to generate and screen cell lines from various plant species, for the production of valuable specialized metabolites, we obtained six lines of fast growing friable calli from the two related species; *A. deliciosa* (kiwifruit tree) and *A. chinensis* (gold kiwifruit tree). Since friable calli disaggregate easily in liquid media, generating fine suspension cultures, they were chosen as promising candidates for cultivation in bioreactors, and were further characterized.

As expected, the six cell lines were able to generate fine and homogeneous suspension cultures in liquid media (data not shown). The metabolomes of their methanol extracts were then analyzed with an untargeted approach, and, quite surprisingly, the Ad-2,3,4 lines were shown to accumulate various quercetin derivatives, which ranged from 5.14 in Ad-4 to 13.26 mg/100 g fresh weight (FW) in Ad-2, corresponding to 146.38 and 419.76 mg/100 g dry weight (DW).

The reports on the production of quercetin and its derivatives by PCTC show large variations in yield depending on the species from which the cell cultures were generated. In general, we can distinguish two types of reports: those in which the basic ability of the cell line to produce quercetin derivatives is reported, and those that show the results following the application of an optimization process. The former demonstrates the productive attitudes of the species, which are under genetic control; the latter explore the ability to push the potential of the species to its maximum.

Within the first group, the amount of quercetin varied from 6.6 and 7.4 mg/100 g DW, reported, respectively in *Tilia americana* [28] and *Clinacanthus nutans* [29] suspension cultures, to the 14.6 and 23 mg/100 g DW found in calli of *Pluchea lanceolata* [30] and *Centella asiatica* [31], and up to 125 mg/100 g DW from cultures of *Helicteres angustifolia* [32]. Interestingly, Cisnero-Torres and coworkers also investigated the quercetin amount in the explants used to generate the calli, finding that quercetin content in leaves of *C. nutans* was slightly higher than calli (12 versus 7.4 mg/100 g DW) [28].

Within the second group, as expected, higher amounts of quercetin were found, indicating that, as is common for other polyphenols, its accumulation in plant cell culture can be boosted by modulation of culture conditions. In this group, the amount of quercetin varied from 42.9, 45.5, 47 and 80 mg/100 g DW in calli of *Gardenia jasmonides* [33], suspension culture of *Polygonum multiflorum* [34] and calli of *Abutilon indicum* [35] and *Allium cepa* [36] to 300, 725, 1650 and 1730 mg/100 g DW from calli of *Dysosma pleiantha* [37] and *Citrullus colocynthis* [38], cell suspensions of *Caesalpinia pulcherrima* [39] and, finally, callus of *Capparis spinosa* [40]. The treatment used to boost quercetin production included feeding with molecular precursors, such as phenylalanine [33,35,38], use of elicitors such as salicylic acid, methyl jasmonate and fungal elicitors [33,34,36,39,40] and medium nutrients such as sucrose, glutamine, casein hydrolysate, coconut water and peptone extract [34,37,39].

The *A. deliciosa* cultures described in this work were able to accumulate quercetin glycosides up to 13.26 mg/100 g FW in Ad-2 cell line, without the application of any boosting technique. In addition, our calculations were carried out based on the fresh weight and not on the dry weight. The determination of the dry weight in our cell lines showed, for the Ad-2 cell line as an example, a water content of 96.84%, and a 31-fold reduction in weight (FW/DW) when 30 day-old calli from medium A were dehydrated. Thus, the normalized data showed a quercetin content of 419.76 mg/100 g DW. These estimates do not exactly reflect the content in the fresh material, mainly because of the possible degradation of part of the metabolites during the drying process. However, even if the methods are not directly comparable, the amount of quercetin detected in the Ad-2 cell line makes it a very attractive and promising biosystem for the industrial production of quercetin. In addition, the Ad-2 line also showed the highest antioxidant activity. Thus, this cell line should be entered into an acceleration program to further increase the amount of quercetin, by optimizing the culture conditions and elicitation.

## 4. Materials and Methods

### 4.1. Generation of the Actinidia Cell Lines

In order to establish cell lines from *Actinidia deliciosa* cv. Hayward and *Actinidia chinensis* cv. Hort16A, 1-year-old plants generated by micropropagation and grown in a greenhouse were used. Four kinds of explant (apical bud, young stem internode, young nodes with lateral meristem and young leaves) were excided from the upper part of the plants. The explants were treated with ethanol 70% (*v*/*v*) for 1 min, followed by 20 min in sodium hypochlorite 1.5% (*v*/*v*). They were rinsed 5 times with sterilized water, cut into about 0.5 cm pieces and strips and explanted on two solid media, A and B, for callus induction (Table 4). The established calli were transferred every 30 days onto a new medium. To determine the dry weight, 500 mg of 30 days-old calli were collected in triplicate from lines Ad-2, 3 and 4 and dried for 20 h in an oven at 80 °C.

### 4.2. Metabolite Extraction

30-day-old calli were collected and powdered in liquid nitrogen using a mortar and pestle. About 150 mg frozen powder was extracted in 900 µL of LC-MS grade methanol (Honeywell, Seezle, Germany). The tubes were vortexed for 30 s and sonicated in an ice-cold water bath at 40 kHz in a Sonica Ultrasonic Cleaner ultrasonic bath (SOLTEC, Milano, Italy) for 15 min. The samples were then centrifuged at 14,000× *g* for 10 min and the supernatant was recovered. For the quantification of quercetin in *A. deliciosa* lines and the execution of FRAP antioxidant assay, the extraction procedure was repeated on the same pellet two more times and all three methanol supernatants from the same sample were joined together. For the analysis, the extracts were diluted 1:10 (*v*/*v*) with LC-MS grade water, passed through Minisart RC4 filters with 0.2 µm pores (Sartorius, Göttingen, Germany) and 1 or 2 µL (as indicated in Figure 2 and Figure 3) was injected in the UPLC-PDA-ESI-QqTOF system for untargeted metabolomics analyses, whereas 4 µL was injected for metabolite quantification.

### 4.3. Metabolite Analysis by UPLC-PDA-ESI-QqTOF

*Actinidia*-diluted extracts were analyzed with UPLC-PDA-ESI-QqTOF. The UPLC consisted of an ACQUITY I CLASS system (Waters, Milford, MA, USA) equipped with a reverse phase BEH C18 column (2.1 mm × 100 mm, 1.7 µm) and maintained at 30 °C. The chromatographic method lasted 20 min and included the following steps: (i) initial condition of 1% B; (ii) isocratic condition at 1% B for 1 min; (iii) gradient to 40% B at 10 min; (iv) gradient to 70% B at 13.5 min; (v) gradient to 99% B at 14 min; (vi) isocratic condition at 99% B for 2 min; (vii) gradient to 1% B at 16.10 min; (viii) isocratic condition at 1% B (re-equilibrium to restore the initial condition) for 3.9 min. The solvents were: (A) water acidified with 0.1% (*v*/*v*) formic acid and (B) acetonitrile. Flux was set at 0.350 mL/min. Samples were placed in the Acquity FTN Autosampler and kept at 8 °C. The UPLC system was linked to an Acquity PDA detector (Waters) followed by a Xevo G2-XS qTOF mass spectrometer (Waters). The PDA carried an eLambda detector able to detect from 190 to 800 nm. The mass spectrometer was equipped with an electrospray ionization (ESI) source operating in either positive or negative ionization modes. The ion source parameters were the same as described in a previous publication [41]. The QqTOF mass spectrometer was set to acquire data in continuum and sensitivity modes. The scan range was set to 50–2000 *m*/*z* and the scan time at 0.3 s. Argon gas was used to perform CID (collision induced fragmentation) and the collision energy was fixed at 35 V. Masslynx v4.1 software (Waters) was used to manage all the UPLC, PDA and ESI/QqTOF instrument functions. In order to check the accuracy of the mass spectrometer, a solution of 100 pg/µL leucine-enkephalin was injected with a flow rate of 10 µL/min and generating a signal of 556.2771 in positive mode and 554.2615 in negative mode. The raw MS data files were processed using Masslynx v4.1 software (Waters).

### 4.4. Metabolite Quantification

The details of quercetin identification are reported in Section 2. Other metabolites were identified by comparing retention time, *m*/*z*, isotopic pattern and fragmentation pattern with those of an in-house library of authentic standards, or by comparison of *m*/*z* and isotopic pattern with those of public databases.

For quantification, peak areas of the quercetin derivatives were obtained from the UPLC-PDA-QqTOF data, using the DAD chromatograms at 355 nm of wavelength, by the automatic integration function available in the software Masslynx v4.1, and each peak was manually checked. Commercial quercetin-3-*O*-glucoside and quercetin-3-*O*-rutinoside (Extrasynthese, Genay, France) were used as outer standards.

### 4.5. FRAP Antioxidant Assay

Antioxidant activity was assessed in vitro using a FRAP assay as previously described [42]. Briefly, 20 µL of each methanolic extract was added to 200 µL of FRAP reagent in a 96-well microplate (Sarstedt, Nümbrecht, Germany) and incubated at 37 °C for 15 min in the dark. Absorbance was recorded at 593 nm with an Infinite200Pro Microplate reader (Tecan Italia, Cernusco sul Naviglio, Italy) and antioxidant activity was expressed as µmol/100 g FW in comparison to a Trolox (Sigma-Aldrich, Saint Louis, MO, USA) calibration curve. Each extract was analyzed in triplicate.

### 4.6. OPLS Correlation Analysis

Raw chromatographic data recorded for Ad samples were processed through Progenesis QI (Waters) to obtain a metabolomics datamatrix. This was added to FRAP datamatrix in SIMCA 13 (Umetrics, Malmö, Sweden) and subjected to orthogonal partial least square (OPLS) analysis to look for linear relationships between antioxidant activity and metabolites of Ad cell lines.

## Figures and Tables

**Table 1:** Retention time (Rt, in minutes), maximum absorbance (λmax), *m*/*z* detected and *m*/*z* expected in negative ionization mode, elemental formula, putative identification, main fragments (ms/ms).

Rt (min)	λ Max (nm)	*m*/*z* (-) Detected	*m*/*z* (-) Expected	Elemental Formula	Putative Identification	ms/ms Fragments
4.64	284.53; 355	463.088	463.088	C_21_H_20_O_12_	Quercetin-*O*-hexoside	301.034
4.64	284.53; 355	465.103	465.103	C_21_H_22_O_12_	Dihydroquercetin-*O*-hexoside (taxifolin-*O*-hexoside)	285.036; 303.050
4.73	249.53; 355.53	625.140	625.140	C_27_H_30_O_17_	Quercetin-*O*-dihexoside	301.034
4.74	253.53; 354.53	771, 1976	771.198	C_33_H_40_O_21_	Quercetin-*O*-hexoside desoxyhexoside *O*-hexoside	299.019; 301.034; 463.086; 609.145; 625.140;
4.74	253.53; 354.53	817.204	817.204	C_33_H_40_O_21_+ HCOOH	Quercetin-*O*-hexoside-desoxyhexoside *O*-hexoside, formic adduct	301.034; 463.086; 609.145; 625.140;
4.83		278.066	278.066	C_13_H_13_NO_6_	Coumaric acid-aspartic acid	163.038 (coumaric acid);
5.06		278.066	278.066	C_13_H_13_NO_6_	(Coumaric acid-aspartic acid)	-
5.13	249.53; 355.53	609.143	609.146	C_27_H_30_O_16_	Quercetin-*O*-hexoside-*O*-desoxyhexoside	299.019; 301.034; 463.084
5.13	249.53; 355.53	577.134	577.134	C_30_H_25_O_12_	Procyanidin dimer P2-type	289.070
5.21	284.532; 355.53	449.109	449.108	C_21_H_22_O_11_	Dihydrokaempferol-*O*-hexoside	287.055; 301.071
5.21	249.53; 355.53	639.156	639.156	C_28_H_32_O_17_	Methyl quercetin-*O*-dihexoside (isorhamnetin-*O*-dihexoside)	315.0504; 463.087
5.45		289.071	289.071	C_15_H_14_O_6_	Epicatechin *	-
5.55		461.165			Ui	-
5.62	249.53; 355.53	771.974	771.198	C_33_H_40_O_21_	Quercetin-*O*-hexoside-*O*-hexoside-desoxyhexoside	301.034; 463.086; 609.454
5.712	249.53; 355	625.140	625.140	C_27_H_30_O_17_	Quercetin-*O*-dihexoside	299.019; 300.026; 301.034; 463.086
6.29	249.53; 354.53	609.147	609.146	C_27_H_30_O_16_	Quercetin-*O*-hexoside desoxyhexoside	300.026; 301.033
6.35	253.53; 355.53	609.147	609.146	C_27_H_30_O_16_	Quercetin-3-*O*-rutinoside *	151.003; 178.997; 299.019; 300.027; 301.033; 609.145
6.51	249.53; 355	463.088	463.088	C_21_H_20_O_12_	Quercetin-*O*-hexoside	300.026; 301.033
6.57	254.53; 355.53	463.088	463.088	C_21_H_20_O_12_	Quercetin-3-*O*-glucoside *	151.003; 178.997; 299.019; 300.027; 301.033
6.82	248.53; 355.53	593.151	593.150	C_27_H_30_O_15_	Quercetin-*O*-di desoxyhexoside	285.038, 301.032
6.93	249.53; 355	623.160	623.161	C_28_H_32_O_16_	Methyl quercetin-*O*-hexoside desoxyhexoside	299.019; 315.049
7.09	249.53; 355.53	447.092	447.092	C_21_H_20_O_11_	Kaempferol-*O*-hexoside	255.029; 284.031; 285.038
7.15	253.53; 355.53	447.093	447.092	C_21_H_20_O_11_	Quercetin-*O*-desoxyhexoside	151.003; 178.997; 299.019; 300.026; 301.033
7.19	249.53; 330.53; 355	477.103	477.066	C_21_H_18_O_13_	Quercetin-*O*-glucuronide	300.026; 301.033; 314.042; 315.047
7.19	249.53; 330.53; 355	709.380			Ui	300.026; 301.033; 314.042; 315.047
7.32	327.35?	515.072			Ui	160.956; 162.950
7.38	249.53; 355	433.113	433.113	C_20_H_18_O_11_	Trihydroxyflavanone-*O*-hexoside (naringenin)	271.060; 301.034
7.38	249.53; 355	463.088			Quercetin-*O*-hexoside	151.003; 178.997; 301.034
7.38	249.53; 355	415.073			Ui	271.060; 301.034
7.67		663,373; 709,379			Ui	160.956; 162.950; 439.320;
8.14		663,373; 709,379			Ui	160.956; 162.950; 439.320;
8.24		711.395	711.408	C_34_H_65_O_13_P	Phosphatidylinositol (25,0)	503.366
8.28		711.395	711.408	C_34_H_65_O_13_P	Phosphatidylinositol (25, 0)	503.367

* These features have been uniquely identified through the use of authentic standards. Ui, Unidentified.

**Table 2:** Quantification of quercetin derivatives in Ad cell lines expressed as mg/100 g fresh weight (FW) and, following normalization for the weight loss during dehydration, as mg/100 g dry weight (DW). Each value is the mean of two technical replicates, +/− standard deviation.

	mg/100 g FW			mg/100 g DW		
Metabolite	Ad-2	Ad-3	Ad-4	Ad-2	Ad-3	Ad-4
Quercetin-3-*O*-rutinoside	3.11 ± 0.15	4.87 ± 0.23	2.67 ± 0.14	94.48 ± 4.88	140.54 ± 6.50	76.01 ± 4.12
Quercetin-3-*O*-glucoside	5.75 ± 0.21	0.81 ± 0.01	0.69 ± 0.09	181.99 ± 6.52	23.58 ± 0.31	19.77 ± 2.46
Other quercetin derivatives	4.40 ± 0.18	4.20 ± 0.13	1.78 ± 0.87	139.29 ± 5.72	121.14 ± 3.62	50.60 ± 24.93
Total quercetin derivatives	13.26 ± 0.23	9.90 ± 0.36	5.14 ± 0.64	419.76 ± 7.37	285.26 ± 10.44	146.38 ± 18.35

**Table 3:** List of Ad-2 metabolites correlating with antioxidant activity.

P[1]	pq(corr)[1]	Correlating Metabolites
0.12	0.999	Epicatechin
0.11	1.000	Quercetin-3-*O*-rutinoside
0.11	1.000	Ui.
0.11	1.000	Procyanidin P2 type
0.49	1.000	Quercetin-*O*-hexoside
0.30	0.999	Quercetin-*O*-desoxyhexoside
0.13	0.999	Ui.
0.15	0.999	Quercetin-*O*-glucuronide
0.21	0.979	Taxifolin-*O*-hexoside

Ui = unidentified.

**Table 4:** Composition of culture media.

Ingredients	Medium A	Medium B	Medium C
salts	MS	MS	MS
sucrose	25 g/L	25 g/L	25 g/L
2.4 D	1 mg/L	-	-
6-BAP	0.5 mg/L	0.5 mg/L	0.25 mg/L
IBA	-	1 mg/L	0.5 mg/L
agar	0.7%	0.7%	0.7%
pH	5.8	5.8	5.8
